# Comparison of the Peak Inspiratory Pressure and Lung Dynamic Compliance between a Classic Laryngeal Mask Airway and an Endotracheal Tube in Children Under Mechanical Ventilation

**Published:** 2017-06

**Authors:** Alireza Mahdavi, Seyed Sajad Razavi, Bita Malekianzadeh, Afsaneh Sadeghi

**Affiliations:** Department of Pediatric Anesthesia, Shahid Beheshti University of Medical Science, Tehran, Iran.

**Keywords:** Dynamic compliance, Inspiratory pressure, Endotracheal tube, Classical laryngeal mask airway

## Abstract

**Background:**

The present study was performed with the aim of comparing the peak inspiratory pressure and lung dynamic compliance between a classic laryngeal mask airway (LMA) and an endotracheal tube in children under mechanical ventilation.

**Materials and Methods:**

In this study, 30 children aged 1 to 7 years with a physical condition of ASA I–II who were admitted for operations to repair inguinal hernias, hydroceles, or hypospadias were randomly enrolled. After induction of anesthesia, the appropriate laryngeal mask was used for each patient and they were placed under pressure-controlled mechanical ventilation. The peak inspiratory pressure was adjusted and recorded to obtain an appropriate tidal volume, then the laryngeal mask was removed and the appropriate size uncuffed endotracheal tube was inserted and the patient was placed again under controlled mechanical ventilation. The required settings were adjusted and peak inspiratory pressure and tidal volume were measured and recorded by the ventilator. Dynamic compliance was also calculated in both cases using the appropriate formula.

**Results:**

The results showed that peak inspiratory pressure (PIP) with the use of LMA in children under mechanical ventilation was less than the PIP with the use of an endotracheal tube (p<0.05). Also, the pulmonary dynamic compliance with a laryngeal mask was greater than the use of an endotracheal tube (p<0.05).

**Conclusion:**

A laryngeal mask airway due to its low airway resistance and high dynamic compliance is an acceptable alternative to a tracheal tube during mechanical ventilation and it can be a good alternative to the endotracheal tube, especially during mechanical ventilation of children, in whom avoidance of barotrauma is desirable.

## INTRODUCTION

Difficulty or inability to perform airway management is still the most important single factor causing morbidity and mortality in patients under general anesthesia (1–3). Supraglottic devices are less invasive than endotracheal tubes and provide a safer airway than a face mask, and they can be used for both spontaneous breathing and positive pressure ventilation. The laryngeal mask airway has been used in routine and difficult airway problems, and it is an important element in the difficult airway algorithm (1). Tracheal intubation in critical patients and in emergency cases is associated with a high prevalence of complications such as esophageal intubation, endobronchial intubation, and aspiration (3). Practical guides have been developed and are recommended for managing difficult airways and to prevent complications (4–8). Classical laryngeal mask airways have been used in more than 200 million patients since 1988. However, lack of proper placement and the resulting aspiration and pneumonia are common complications of this modality.

The laryngeal mask airway (LMA) is the best supraglottic device currently used in airway management and comes in sizes 1–6 for use from neonates to adults with more than 100 kg of weight (6). Also, LMA is able to provide airway management in infants and children. Its use is contraindicated in patients with a risk of aspiration of gastric contents, but if it is chosen and placed correctly and mechanical ventilation is adjusted with positive pressure volumes (PPV) less than 10 mg/kg, almost no episodes of gastric distention will be observed. Safe use of PPV with LMA at different ages has been confirmed (9). Compared to the endotracheal tube, LMA has advantages such as less manipulation of the airway and easier application and it is a good alternative to the endotracheal tube, especially in short-term surgery. LMA is less invasive and results in less postoperative discomfort. In addition, during its use, there are significantly fewer hemodynamic changes compared to the use of endotracheal tubes (ETT) (10).

In a comparative study, it was shown that LMA provides appropriate airway management in more than 90% of infants and children. No differences were observed between LMA and the endotracheal tube in complications such as laryngitis, bronchospasm, or increased saliva. Coughing and holding the breath were observed at lower rates in the use of LMA (9). In a study of children aged 2–10 years (with a weight of 10–20 kg), in order to assess breathing with positive pressure and the use of LMA or ETT, it was observed that application of the LMA was easier than inserting an ETT, and hemodynamic changes, airway complications, and soft tissue trauma were significantly less with the use of LMA (10). In another study on adults under undergoing orthopedic surgery that was performed in Iran, it was found that LMA creates higher resistance and lower dynamic compliance than ETT (11). In the studies performed, it was specified that in the case of mechanical ventilation with the LMA, the inspiratory pressure should be less than 20 cm H_2_O; if it is higher, the risk of inflation of the stomach and regurgitation increase, especially when the LMA is not inserted correctly, and this occurs more frequently with the use of size 1 and 1.5. Compared to the controlled volume mode, pressure controlled ventilation reduces the required inspiratory pressure and it can improve ventilation distribution in infants and children (12). Also, in a comparative study on mechanical ventilation with LMA and an uncuffed endotracheal tube in children weighing less than 30 kg, in the cases of older children, LMA with a size larger than 2.5 was inserted and air leaks compared between LMA and ETT were similar, but it was not recommended to use LMA in children weighing less than 10 kg (13).

Today, reducing airway resistance and improving dynamic compliance during mechanical ventilation are desirable goals to control hemodynamic status and to prevent pulmonary barotrauma, especially in children (11). Therefore, it is necessary to work toward the use of lower inspiratory pressure and airway resistance and higher compliance during mechanical ventilation in children. Since few studies have been conducted in this area and different results have been obtained from studies in adults, the present study was conducted on children aged 1 to 7 years with the aim of comparing two airway management devices (LMA and tracheal tubes) in each patient and with the use of required parameters and calculation of lung compliance, in order to identify the device with fewer side effects on respiratory function.

## MATERIALS AND METHODS

Thirty ASA I–II pediatric patients were studied, ranging in age from 1 to 7 years old. They had all been scheduled for inguinal hernia, hydrocele, or hypospadias elective repair surgery. The required sample size was estimated at 30 persons according to the formula used for comparing the means. After initial examination before surgery and an explanation about the anesthesia method, the methods used to manage the airways and ventilation of the patient during surgery, and the goal to determine the effects and advantages of a laryngeal mask, informed consent was obtained from the patients’ parents for their participation in this research project. Inclusion criteria were the absence of obesity (BMI < 30), no history of a difficult airway or obstructive airway disease, and the absence of an active respiratory infection. The use of an LMA is associated with an increased risk of complications of the airway such as airway obstruction and more leakage and higher pressure required in age under one year.

During the operation, the patient was placed in the supine position after intravenous induction, using propofol 3–2 mg/kg, fentanyl 1–2 μg/kg, and atracurium 0.5 mg/kg to create adequate muscle relaxation during the operation; after three minutes, the LMA in the appropriate size was inserted. In order to maintain anesthesia, isoflurane and N_2_O/O_2_ were prescribed in a 50:50 ratio and the patient was placed under mechanical ventilation in pressure controlled ventilation mode and the pressure was adjusted to obtain TV = 7–10 cc/kg and the RR was set according to the patient’s age and PETCO_2_. Ten minutes later and after stabilizing the patient’s condition, peak inspiratory pressure and tidal volume measured by the ventilator were recorded. Then the LMA was replaced by an endotracheal tube of the appropriate size, and the endotracheal tube was fixed in a convenient location. The parameters of the ventilator were adjusted again and the peak inspiratory pressure and TV were recorded 10 minutes later. The endotracheal tubes and LMAs were inserted in all patients by an expert anesthesiologist. The information was recorded and collected in the patient’s form. Dynamic compliance in both situations for each patient was calculated using the following formula.

$$\frac{\text{TV}}{\text{PIP}-\text{PEEP}}$$

After data collection, the data were statistically analyzed by SPSS 21 software and all of the quantitative variables were expressed as mean ± standard deviation and all of the qualitative variables were expressed as number (percentage). A t-test was used to compare the quantitative variables between the two groups.

## RESULTS

In the present study, 30 boys with a mean age of 2.98 ± 1.23 and mean weight of 13.78 ± 3.20 kg were studied. LMA size 2 was used for 26 patients (86.7%) and LMA size 2.5 was used for 4 patients (13.3%). The endotracheal tubes sized 4, 4.5, 5, and 5.5 were used for 1 (3.3%), 14 (46.7%), 11 (36.7%), and 4 patients (13.3%), respectively.

The results of the statistical test indicate that there was a significant relationship between the use of a tube and peak inspiratory pressure (PIP) and that the PIP with the use of LMA in children under mechanical ventilation was less than the PIP with the use of an endotracheal tube (P < 0.05) (Table 1).

**Table 1.** Mean±standard deviation of the variables measured and compared using t-test

**Variable**	**Group**		**P-Value**
	**LMA**	**ETT**	
Peak inspiratory pressure (PIP)	10.40±1.19	13.20±1.95	0.002^*^
Tidal volume	140.81±30.25	139.67±28.95	0.535^NS^
Pulmonary dynamic compliance	13.62±3.03	10.84±2.98	0.001^*^

NS: Nonsignificant

* P<0.05

Also, the results showed that lung dynamic compliance with the use of LMA was greater than the lung dynamic compliance with the use of an endotracheal tube (P < 0.05) (Table 1).

## DISCUSSION

LMA is an appropriate non-invasive alternative to an endotracheal tube, and it is an acceptable method during a short-term operation and in cases where intubation is difficult. Because there is no need for laryngoscopy, it does not have many adverse consequences related to its use. Furthermore, it has advantages such as less manipulation of the airway and easier insertion compared with an endotracheal tube. The studies showed that the hemodynamic changes during LMA insertion were significantly less than that for ETT (3). On the other hand, reduced airway resistance and improved dynamic compliance in the intensive care unit or during general anesthesia, especially in patients with pulmonary problems, is considered to be an important goal.

In the present study, the results of statistical testing showed that PIP with the use of LMA in children under mechanical ventilation was less compared with the use of an endotracheal tube and lung dynamic compliance with the use of LMA in children was greater compared with the use of an endotracheal tube. In each patient using ETT, there was a need to set the peak pressure at higher values than for LMA.

The significant difference between airway resistance and dynamic compliance in the present study confirms that in general, PIP with the use of LMA in children under mechanical ventilation is less compared with the use of an endotracheal tube and this means that the use of LMA is acceptable because it reduces airway resistance and improves pulmonary compliance during mechanical ventilation, controls hemodynamic status, and eventually, prevents pulmonary barotrauma, which are all very important in children (2). On the other hand, in patients with pulmonary parenchymal disease, more pressure should be applied to obtain the desired tidal volume. LMA creates higher dynamic compliance and it is thought to be a proper airway device in these patients.

On the other hand, with increased pulmonary compliance, there is less trans-pulmonary pressure to deliver tidal volume to the lungs. Therefore, increased pulmonary compliance decreases PIP and in fact, increased pulmonary compliance decreases the work of breathing and increases the chances of success in separating the patient from the ventilator. In this regard, Ozden et al. compared LMA and a tube without a cuff in infants in terms of postoperative airway complications. The results showed that LMA is a more appropriate device to be used in infants compared with an endotracheal tube. It requires less manipulation of the airway and results in a lower incidence of laryngitis (14).

Tulgar et al. compared 4 pediatric groups that underwent laparoscopic surgery with either ETT + muscle relaxant (MR), ETT without MR, LMA with a subparalytic dose of MR, and LMA without MR. Anesthesia and recovery time were significant longer in ETT in the muscle relaxant group. There was no significant difference between basal intragastric pressure, average intragastric pressure, and average peak airway pressure during insufflation (15).

In another study on 60 children under general anesthesia, the respiratory parameters during ventilation with positive pressure with LMA vs an endotracheal tube were compared with each other. No differences were observed between the two groups in terms of inspiratory pressure, tidal volume, or leakage of gas but a significant difference was observed between them in terms of airway resistance and pulmonary compliance, and LMA was superior. This result is consistent with the results of the present study (16).

Idrees and Khan also compared LMA and endotracheal tubes during mechanical ventilation in adults undergoing peripheral limb surgery. The results showed that the hemodynamic changes during insertion of the tube with the LMA was lower but no significant difference was observed in terms of cardiac effects during extubation. The incidence of cough and mild hypoxemia during extubation with the endotracheal tube was higher. Therefore, an LMA is more appropriate for mechanical ventilation in selected patients (17).

Hashemian et al. compared i-gel and classic LMA in paralytic anesthetized patients. The device insertion parameters, some ventilatory parameters, and adverse effects were studied. The duration of the insertion time was significantly different between the two groups. The results showed i-gel can be an alternative to classic LMA for controlled ventilation during anesthesia. Their results are in general agreement with the present study (18).

According to the results of the Asida and Ahmed study, the use of LMA is a reliable ETT alternative in pediatric patients due to its low failure rate and ease of insertion (19).

In another study on adults undergoing orthopedic surgery, which was performed in Iran, it was found that an LMA creates higher resistance and lower dynamic compliance than ETT (11). This result is inconsistent with the results of the present study. The advantages of LMA in children aged under 12 years were investigated and compared to ETT. The results of 16 clinical trial studies were analyzed. The results showed that compared to ETT, LMA has three advantages: a lower incidence of cough while awake, a lower prevalence of sore throat and nausea after surgery, and no significant difference was observed between them in terms of laryngitis and bronchospasm (20). Also, in another study by Genzwuerker et al., LMA was introduced as an effective device in airway management. The results of both studies are consistent with the results of the present study (21).

It seems that the reasons for the inconsistency in the results of some studies are the duration of anesthesia and the studied population. Our study has some limitations. The studied children were ASA I–II and therefore our results cannot be extended to patients with previous respiratory disease or obesity. In addition, the duration of surgery was short.

## CONCLUSION

It can be concluded that LMA is an acceptable alternative to an endotracheal tube, especially during short-term operations in children due to lower airway resistance and higher dynamic compliance, and prevention of intubation complications such as cough, less stress on the patient, and as a result, better control of the patient’s hemodynamic responses (22).

Therefore, we suggest future studies examine the use of LMA in various surgical procedures with a longer duration and be performed in various positions, and also in patients with pulmonary disease and in the intensive care unit.
